# Ciliary beat pattern analysis below 37°C may risk PCD misdiagnosis

**DOI:** 10.1186/2046-2530-1-S1-P26

**Published:** 2012-11-16

**Authors:** C Jackson, PM Goggin, JS Lucas

**Affiliations:** 1 Primary Ciliary Dyskinesia Group, University of Southampton Faculty of Medicine, Southampton NIHR Respiratory Biomedical Research Unit, University Hospital Southampton Foundation Trust, UK

## 

Primary Ciliary Dyskinesia (PCD) is a rare inherited multi-genic disorder of mucociliary function. Patients with indicative clinical profiles referred to the UK specialist PCD service receive a diagnosis based on multiple factors. These include high-speed video microscopy (HSVM) analysis of ciliary beat pattern (CBP) and ciliary beat frequency (CBF) at 37°C (for in vivo modelling). In PCD, ciliary axonemal defects generate abnormal CBP with/without abnormal CBF. Corresponding and predominant ultrastructural defects are determined by TEM, except in atypical cases. We report an atypical PCD patient (8 months old) with respiratory and nasal symptoms since birth, situs inversus and serous otitis media. HSVM confirmed abnormal and hyperfrequent ciliary function at 37°C on four occasions, but normal ciliary ultrastructure. On two occasions CBP and CBF (mean ±SD) were assessed at 37oC and room temperature (21-24°C). At 37°C CBF was hyperfrequent (34.4 Hz ±13.5 n=11; 26.3 Hz ±3.4 n=6) and CBP consistently abnormal with interrupted, short range, dyskinetic motility. However at room temperature the same cilia reverted to CBF (15.2 Hz ±4.5 n=2; 12.6 Hz ±0.8 n=6) within our normal range (11-20 Hz) with improved ciliary coordination and range of movement, suggesting a PCD variant with temperature sensitive CBP. Recent research suggests that healthy human epithelium maintains a normal CBP at temperatures as low as 2°C, and low temperature ciliary analysis may diagnostically replace HSVM. However, in light of our case study we conclude that temperature sensitive variants of PCD may exist and CBP analysis below 37°C without HSVM may risk PCD misdiagnosis.

